# PKA mediates modality-specific modulation of the mechanically gated ion channel PIEZO2

**DOI:** 10.1016/j.jbc.2023.104782

**Published:** 2023-05-04

**Authors:** Irina Schaefer, Clement Verkest, Lucas Vespermann, Thomas Mair, Hannah Voß, Nadja Zeitzschel, Stefan G. Lechner

**Affiliations:** 1 Institute of Pharmacology, Heidelberg University, Heidelberg, Germany; 2 Department of Anesthesiology, University Medical Center Hamburg-Eppendorf, Hamburg, Germany; 3 Section for Mass-Spectrometry and Proteomics, University Medical Center Hamburg-Eppendorf, Hamburg, Germany

**Keywords:** PIEZO2, mechanotransduction, PKA, pain, post-translational modification

## Abstract

PKA is a downstream effector of many inflammatory mediators that induce pain hypersensitivity by increasing the mechanosensitivity of nociceptive sensory afferent. Here, we examine the molecular mechanism underlying PKA-dependent modulation of the mechanically activated ion channel PIEZO2, which confers mechanosensitivity to many nociceptors. Using phosphorylation site prediction algorithms, we identified multiple putative and highly conserved PKA phosphorylation sites located on intracellular intrinsically disordered regions of PIEZO2. Site-directed mutagenesis and patch-clamp recordings showed that substitution of one or multiple putative PKA sites within a single intracellular domain does not alter PKA-induced PIEZO2 sensitization, whereas mutation of a combination of nine putative sites located on four different intracellular regions completely abolishes PKA-dependent PIEZO2 modulation, though it remains unclear whether all or just some of these nine sites are required. By demonstrating that PIEZO1 is not modulated by PKA, our data also reveal a previously unrecognized functional difference between PIEZO1 and PIEZO2. Moreover, by demonstrating that PKA only modulates PIEZO2 currents evoked by focal mechanical indentation of the cell, but not currents evoked by pressure-induced membrane stretch, we provide evidence suggesting that PIEZO2 is a polymodal mechanosensor that engages different protein domains for detecting different types of mechanical stimuli.

PIEZO1 and PIEZO2 are mechanically gated ion channels that enable cells to detect and respond to mechanical stimuli in order to maintain cell, tissue, and not least body integrity in an ever-changing mechanical environment (1, 2). Some tumor cells, for example, utilize PIEZO2 to detect relatively small and slowly generated traction forces that occur during cell migration (3), whereas sensory neurons from the dorsal root ganglia (DRG) require PIEZO2 to detect tactile stimuli and comparably large noxious mechanical stimuli impinging on the skin (4, 5, 6, 7, 8). Moreover, PIEZO2 is required for the detection of bladder distension (9), airway stretch (10), and muscle tension (11, 12). The fact that PIEZO2 can detect mechanical stimuli of different origins with a wide range of magnitudes and dynamics raises the question as to whether cells can adjust PIEZO channel sensitivity in order to reliably detect the type of mechanical stimulus they encounter. Indeed, it was shown that different splice variants with slightly yet significantly different properties are expressed in the urinary bladder, the lung, and within sensory ganglia (13). Moreover, several protein interaction partners that can enhance (STOML3, EPAC1, and TMEM150c (14, 15, 16, 17)) or inhibit (*e.g.*, MTMR2, SERCA2, Annexin A6, and Nedd4-2 (18, 19, 20, 21)) PIEZO2 sensitivity have been described.

In addition to being modulated by alternative splicing and interaction with other proteins, most ion channels are also subject to post-translational modifications, such as phosphorylation, which can further fine-tune channel function (22, 23, 24, 25, 26). Whether PIEZO2 function is also regulated by post-translational modifications, which would allow cells to quickly adjust their mechanosensitivity in order to adapt to a changing environment, is, however still unclear. A prominent example of a relatively rapid change in mechanosensitivity is the sensitization of nociceptive sensory afferents by inflammatory mediators, many of which activate G protein–coupled receptors. We have previously shown that the algogens ATP and UTP sensitize so-called rapidly adapting (RA) mechanotransduction currents in a subset of nociceptive DRG neurons, which were later shown to be mediated by PIEZO2, *via* activation of the G_q_-coupled P2Y_2_ receptor (27). Moreover, we recently found that RA-mechanotransduction currents in peptidergic nociceptors are also enhanced by an inflammatory “soup”, which consisted of bradykinin, prostaglandin E_2_, serotonin, and histamine (28). Recently, Del Rosario *et al.* (29) showed that PIEZO2-dependent mechanotransduction currents in DRG neurons are modulated by G_i_-coupled receptors in a G_βγ_-dependent manner. Finally, Dubin *et al.* (30) demonstrated that the sensitizing effect of bradykinin on RA currents is mediated by the B_2_ receptor in a PKA- and PKC-dependent manner and further showed that direct activation of PKA and PKC in DRGs and human embryonic kidney 293 cells also potentiates PIEZO2-mediated currents. The mechanisms underlying PKA- and PKC-dependent modulation of PIEZO2 are however still elusive.

Hence, we here set out to examine the molecular mechanism underlying PKA-dependent modulation of PIEZO2, using site-directed mutagenesis of putative PKA phosphorylation sites on PIEZO2 and patch-clamp recordings. We focused on the mechanism of PKA-dependent modulation because PKA is a downstream effector of many inflammatory mediators that induce mechanical pain hypersensitivity (31). Our data suggest that the PKA-dependent modulation of PIEZO2 requires multiple serines that are located on different intracellular domains. Moreover, we demonstrate that PKA activity only modulates PIEZO2 currents that are elicited by focal mechanical stimulation, that is, poking of the cell surface with a small glass rod, but not currents that are evoked by membrane stretch in so-called pressure-clamp recordings. The observation that poking- and stretch-evoked currents can be modulated independent of each other is conceptually very interesting as it supports recent studies from others and us, which had suggested that poking and stretch activate PIEZO2 and PIEZO1 *via* different intraprotein force transmission pathways (32, 33, 34).

## Results

### Poking- but not stretch-evoked PIEZO2 currents are potentiated by PKA

**Figure 1:**
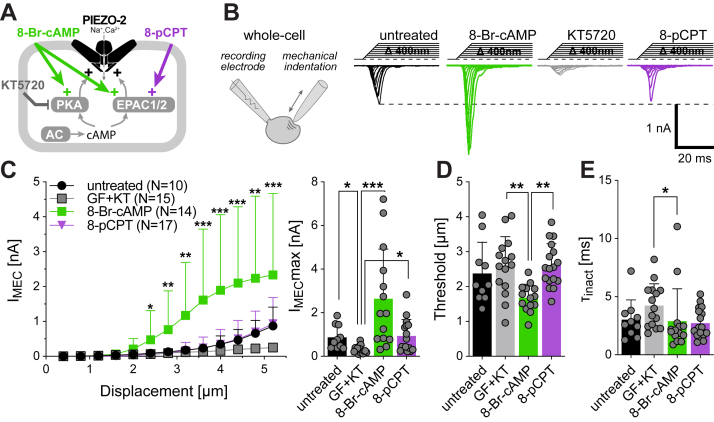
**Poking-evoked PIEZO2-mediated whole-cell currents are regulated by PKA.***A*, schematic representation of the possible cAMP-dependent cellular signaling cascade (PKA or EPAC1/2) potentiating PIEZO2, in addition to their respective pharmacological modulators used throughout the study. *B*, *cartoon* depicting the recording paradigm (*left*) and representative PIEZO2 whole-cell currents (holding −60 mV) evoked by increasing mechanical indentation of N2a-P1KO cells (*right*) in the absence (*black*) or in the presence of the PKA activator 8-bromo-cAMP (*green*), the PKA and PKC inhibitors GF and KT (*gray*) and of the EPAC1/2 activator 8-pCPT (*purple*). *C*, displacement–response curves (*left*) and scatter plot (*right*) of maximal peak current amplitudes of poking-evoked currents recorded from N2a cells expressing PIEZO2 and treated with the indicated drugs. Data are presented as the mean ± SD. Number of cells per group is indicated in the legend. Comparison with Kruskal–Wallis test *p* < 0.0001 and Dunn’s post-test *p* < 0.05 ∗, *p* < 0.001 ∗∗, *p* < 0.0001 ∗∗∗, 8-Br-cAMP *versus* untreated. See also Fig. S1. *D*, mechanical activation thresholds of PIEZO2 whole-cell currents from treated and untreated cells. Data are presented as the mean ± SD with individual values. Untreated N = 10, GF + KT N = 11, 8-Br-cAMP N = 14, and 8-pCPT N = 17. Comparison with one-way ANOVA *F*(3, 52) = 5.69, *p* = 0.0019 and Tukey’s post-test, KT *versus* 8Br *p* = 0.0049 and 8Br *versus* 8pCPT *p* = 0.0029. *E*, inactivation time constants (τ_inact_) of PIEZO2 whole-cell currents from treated and untreated cells. Data are presented as the mean ± SD with individual values. Untreated N = 10, GF + KT N = 13, 8-Br-cAMP N = 16, and 8-pCPT N = 19. Comparison with Kruskal–Wallis test *p* = 0.0251 and Dunn’s post-test, *p* = 0.023 KT *versus* 8Br. See also Fig. S1. 8-Br-cAMP, 8-bromo-cyclic-AMP.

To examine the molecular mechanism underlying PKA-dependent modulation, we expressed PIEZO2 in Neuro2a-PIEZO1-KO cells, hereafter referred to as N2a cells, which completely lack endogenous mechanotransduction currents (33, 35, 36). Unless otherwise stated, PIEZO2-mediated currents were elicited by mechanical indentation of the cell membrane with a small glass rod and were recorded in the whole-cell patch-clamp configuration—a commonly used technique referred to as the “poking” technique (37). We first asked to which extent PIEZO2 currents from untreated N2a cells are subject to modulation by basal PKA and PKC activity. To this end, we compared PIEZO2 currents from untreated cells with currents recorded from cells that were incubated with the PKA inhibitor KT5720 (1 μM) or the PKC inhibitor GF109203X (1 μM), or both, for 12 h. Both PKA and PKC inhibition significantly reduced the amplitudes of PIEZO2-mediated currents by approximately 50%, but there was no additive or synergistic effect when both kinases were inhibited simultaneously (Fig. S1, *A* and *B*). Moreover, we observed small but statistically significant effects of PKA inhibition on the inactivation kinetics and the mechanical activation threshold of PIEZO2 (Fig. S1, *C* and *D*). Hence, our data showed that PIEZO2-mediated currents are significantly potentiated by basal PKA and PKC activity. To minimize interexperimental variability caused by possible varying basal PKC activity, all subsequent experiments were performed in the presence of the PKC inhibitor GF109203X. To determine the full extent of PKA-dependent modulation, we next compared PIEZO2 currents from untreated cells with currents recorded from cells that were either pretreated KT5720 for 12 h or incubated with KT5720 for 11 h followed by a 1 h incubation with the PKA activator 8-bromo-cyclic-AMP (8-Br-cAMP, 300 μM) prior to recording (Fig. 1*A*). Strikingly, 8-Br-cAMP treated cells exhibited very large PIEZO2-mediated mechanotransduction currents that were significantly bigger and had significantly lower mechanical activation thresholds than currents recorded from KT5720-treated cells (threshold KT5720, 2.59 ± 0.84 μm *versus* 8-Br-cAMP, 1.68 ± 0.38 μm; mean ± SD, Fig. 1, *B*–*D*). The inactivation kinetics of currents recorded from untreated and 8-Br-cAMP-treated cells were indistinguishable, but those from KT5720-treated cells were significantly slower (Fig. 1*E*). 8-Br-cAMP does, however, not only activate PKA but also EPAC1 (Fig. 1*A*)—that is, exchange protein directly activated by cAMP—which was previously shown to potentiate PIEZO2 currents (15). Hence, to test if EPAC1 activation contributes to the effect of 8-Br-cAMP treatment, we next examined the effect of the Epac-selective cAMP analog 8-pCPT. PIEZO2 currents from cells that were incubated with 10 μM 8-pCPT for 12 h were indistinguishable from currents recorded from untreated cells (Fig. 1, *B*–*E*), indicating that EPAC1 does not contribute to the 8-Br-cAMP-induced potentiation of PIEZO2 activity and is probably not present in N2a cells.

**Figure 2:**
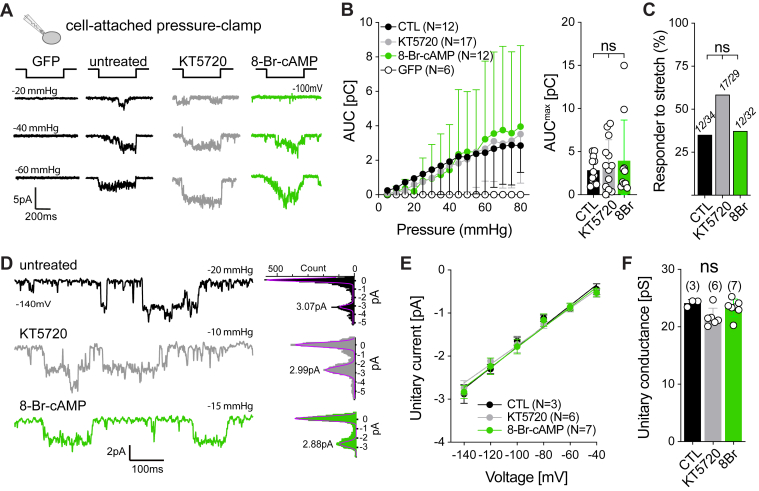
**PIEZO2-dependent stretch-activated currents are not regulated by PKA.***A*, *cartoon* depicting the recording paradigm (*left*) and representative PIEZO2 stretch-activated current (*right*) evoked by increasing negative pressure pulses applied to N2a-P1KO cell patches in the absence (*black*) or the presence of the PKA inhibitor KT5720 (*gray*) and the PKA activator 8-Br-cAMP (*green*). See Fig. S2 for additional example traces. *B*, pressure–response curves (*left*) and scatter plot (*right*) of the maximal total charge transfer (area under the curve [AUC]) evoked by negative pressure of GFP empty vector or PIEZO2 from treated and untreated cells. Data are presented as the mean ± SD. Number of cells per group is indicated in the legend. Comparison with two-way ANOVA *p* = 0.7147. *C*, proportion of cells responding to pressure-induced membrane stretch amongst treated or untreated cells. The numbers of tested cells are indicated within the bars. Number of responders correspond to the ones indicated in the legend of (*B*). Fisher’s exact test, *p* = 0.0894 CTL *versus* KT, *p* = 0.7753 CTL *versus* 8Br, *p* = 0.1269 KT *versus* 8Br. *D*, example traces (*left*) of stretch-activated PIEZO2 currents from untreated (*black*), KT5720-treated (*gray*), and 8-Br-cAMP-treated (*green*) cells and shown with their corresponding current amplitude distribution histograms (*right*). The peak values of Gaussian fits (*purple*) representing the unitary current are indicated. *E*, linear regression fits of the I/V plots (unitary currents *versus* holding potential) of PIEZO2-treated and PIEZO2-untreated cells. Data are presented as the mean ± SD. Number of cells per group is indicated in the legend. *F*, unitary conductance of PIEZO2 in treated and untreated cells. Data are presented as the mean ± SEM with individual values. Number of cells per group is indicated above each bar. Comparison with Kruskal–Wallis *p* = 0.1341. 8-Br-cAMP, 8-bromo-cyclic-AMP.

Another experimental technique, which is commonly used to asses PIEZO channel function, is the so-called pressure-clamp technique. Here, PIEZO currents are recorded in the cell-attached mode of the patch-clamp technique and are activated by stretching the membrane inside the patch-pipette by application of negative pressure (37). We have recently shown that cell poking and membrane stretch activate PIEZO2 *via* different force-transmission pathways (33). Hence, we next asked if PKA equally effectively modulates stretch-activated PIEZO2 currents in pressure-clamp recordings. Strikingly, neither inhibition nor activation of PKA altered stretch-activated PIEZO2 currents. Thus, the pressure–response curves and the proportion of cells responding to pressure stimulation of the currents recorded from untreated, KT5720-, and 8-Br-cAMP-treated cells were indistinguishable from each other (Figs. 2, *A*–*C* and S2, *A*–*C*). We also analyzed single-channel openings evoked by small pressure pulses and found that PKA activity does not alter single-channel conductance (untreated, 24.13 ± 0.61 pS; KT5720, 21.65 ± 1.59 pS; and 8-Br-cAMP, 23.29 ± 1.53 pS; mean ± SD, Fig. 2, *D*–*F*), demonstrating that changes in single-channel conductance do not contribute to the PKA-dependent modulation of PIEZO2.

Taken together, our data show that poking- and stretch-evoked PIEZO2 currents are differentially modulated by PKA. This observation is consistent with our previous work, which suggested that poking and stretch activate PIEZO2 *via* two different mechanisms that can be modulated independent of one another. Moreover, our data show that basal PKA activity has relevant effects on PIEZO2 activity, which might, at least partly, account for interexperimental variability when recording currents from different cell passages or different cell lines.

### Substitution of PKA sites on individual intracellular domains does not abolish PKA-dependent modulation of PIEZO2

**Figure 3:**
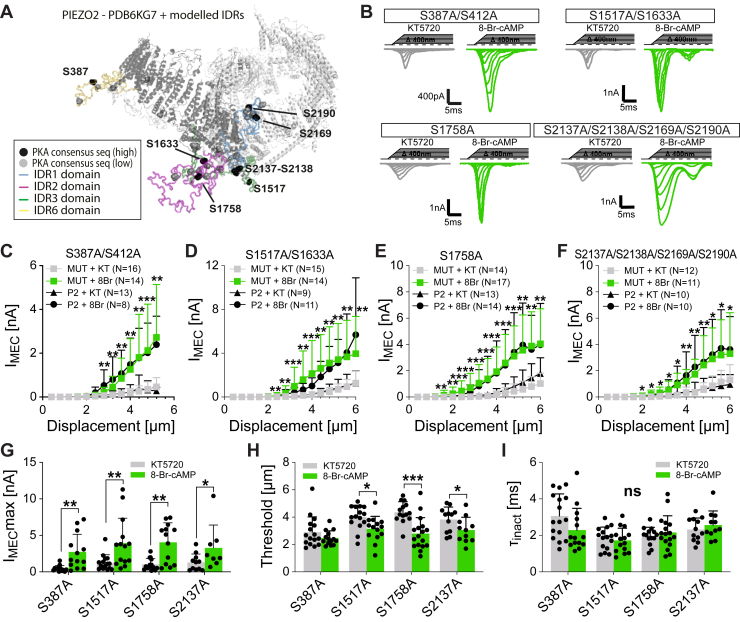
**Mutation of predicted PKA sites from individual intracellular domain of PIEZO2 does not prevent PKA-dependent modulation.***A*, side view of the mouse PIEZO2 structure (Protein Data Bank ID: 6KG7) with the modeled intracellular disordered loops that contained the predicted high (*black spheres*) and low (*gray spheres*) scores. PKA phosphorylation sites colored in the indicated color. See also Table S1. *B*, representative example traces from the different PIEZO2 mutants S387A/S412A (*top left*), the S1517A/S1633A (*top right*), the S1758A (*bottom left*), and the S2137A/S2318A/S2169A/S2190A (*bottom right*) in the presence of the PKA inhibitor KT5720 (*gray*) and the PKA activator 8-Br-cAMP (*green*). *C*–*F*, displacement–response curves of peak current amplitudes of PIEZO2 (*black*) and the different PIEZO2 PKA site mutants (*gray* and *green*) for cells treated with PKA inhibitor or activator. Data are presented as the mean ± SD. Number of cells per group is indicated in the legend. Comparison with Mann–Whitney test *p* < 0.05∗, *p* < 0.001∗∗, *p* < 0.0001∗∗∗, mutant + KT *versus* mutant + 8Br. *G*–*I*, comparison of the mean ± SD maximal current amplitude (*G*), mechanical activation thresholds (*H*), and inactivation time constants (*I*) of whole-cell currents from PIEZO2 mutants (same mutants as in *B*–*F*, labels only contain first mutation for better reading) treated with KT5720 (*gray*) or 8-Br-cAMP (*green*). *Black circles* represent individual data points. Number of cells per group are identical to those in *C*–*F*. Thresholds of the S387A/S412A mutant were compared with Mann–Whitney test *p* = 0.3550 and with Student's *t* test for all other mutants (∗*p* = 0.0161, S1517A; ∗∗∗*p* = 0.00017, S1758A; *p* = 0.0560, S2137A). 8-Br-cAMP, 8-bromo-cyclic-AMP.

Numerous ligand- and voltage-gated ion channels, such as KCNQ channels, Nav1.7, or L-type calcium channels, just to name a few, are modulated by direct phosphorylation by PKA (23, 24, 26). To test if PIEZO2 might also be subject to direct phosphorylation by PKA, we screened the amino acid sequence of PIEZO2 for putative PKA consensus motifs using the Netphos3.1 and the GPS phosphorylation site prediction algorithms (38, 39). This analysis identified a total of 28 putative PKA phosphorylation sites that were localized on intracellular domains of PIEZO2 and are thus likely to be accessible for PKA (Table S1). To further narrow down the list of candidate sites, we excluded all PKA sites that were only predicted by one of the two algorithms and that had a Netphos3.1 score smaller than 0.6. This filtering step reduced the list to a total of eight potentially relevant putative PKA phosphorylation sites (Table S1), of which four are located on the intrinsically disordered intracellular domain that we had previously termed IDR1, two are located on IDR3 and one on each of the domains IDR2 and IDR6 (Fig. 3*A*). To test if these putative phosphorylation sites are required for the PKA-dependent modulation of PIEZO2, we generated channel mutants in which all serines that could potentially be phosphorylated and that were located on the same IDR domain were substituted by alanines. On IDR6, we in addition substituted S412, which was not predicted by the GPS algorithm but had a relatively high Netphos3.1 score. The function of all mutants was assessed with the poking technique in the presence of fully activated PKA (8-Br-cAMP) and in the absence of PKA activity (KT5720). Since we had noticed that the amplitudes of PIEZO2 currents can vary between different cell passages and transfections and that the magnitude of PKA-dependent modulation can also slightly vary between recording sessions, we performed separate control recordings with wildtype PIEZO2 in each session. Surprisingly, all four PIEZO mutants were modulated to the same extent as PIEZO2 wildtype. Thus, the current amplitudes of the mutant channels recorded in the presence of KT5720 or 8-Br-cAMP were identical to the amplitudes of PIEZO2 wildtype currents recorded under the same conditions on the same day (KT5720, *gray squares versus black triangles* and 8-Br-cAMP, *green squares versus black circles* in Fig. 3, *C*–*G*). Moreover, 8-Br-cAMP treatment reduced the mechanical activation thresholds of all tested mutants, except the S387A/S412A double mutant (Fig. 3*H*), whereas the inactivation kinetics were not affected (Fig. 3*I*).

### Deletion of the intracellular domain IDR5 abrogates PKA-mediated modulation of PIEZO2

**Figure 4:**
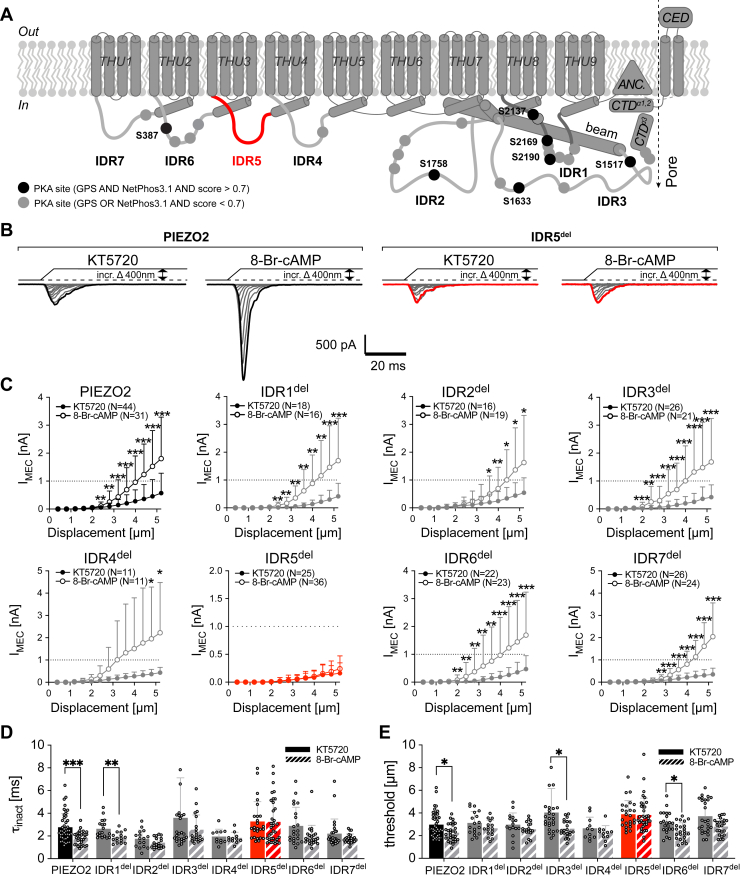
**IDR5**^**del**^**-mediated currents are not modulated by PKA.***A*, topological representation of PIEZO2 with its major domains, its intracellular intrinsically disordered regions (IDR1–7), and the location of the predicted PKA phosphorylation sites (*black* and *gray circles*). *B*, representative example traces of whole-cell current evoked by increasing mechanical indentation from PIEZO2 (*left*) and the previously characterized IDR5^del^ PIEZO2 mutant (*right*) in the presence of the PKA inhibitor KT5720 or the PKA activator 8-bromo-cAMP. C, displacement–response curves of peak current amplitudes of PIEZO2 and the indicated IDR^del^ mutants after inhibition and activation of PKA. Data are presented as the mean ± SD. Number of cells per group is indicated in the legend. Comparison with Mann–Whitney test, *p* < 0.05∗, *p* < 0.001∗∗, *p* < 0.0001∗∗∗ 8-Br-cAMP *versus* KT. *D* and *E*, inactivation time constant (τ_inact_) (*D*) and mechanical activation thresholds (*E*) of whole-cell current from PIEZO2 and IDR^del^ mutants treated with KT5720 (*filled bars*) or 8-Br-cAMP (*dashed bars*). Data are presented as the mean ± SD with individual values. Number of cells per group are identical to those in (*C*). Inactivation time constants and activation thresholds of KT5720- and 8-Br-cAMP-treated cells were compared pairwise for each mutant using Mann–Whitney test. τ_inact_: PIEZO2, *p* = 0.000081; IDR1^del^*p* = 0.0020. Activation threshold: PIEZO2, *p* = 0.004; IDR3^del^*p* = 0.0018; IDR6^del^*p* = 0.012. 8-Br-cAMP, 8-bromo-cyclic-AMP.

The lack of a detectable difference in PKA-dependent modulation between wildtype PIEZO2 and the mutant channels prompted us to question if the Netphos3.1 score cutoff of 0.6 that we had used to narrow down the list of potentially important PKA sites was maybe a bit too stringent. To test if any of the putative PKA sites with low prediction scores or sites that were only predicted by one of the two algorithms, most of which are located on the intracellular IDR domains (Table S1 and Fig. 4*A*), are required for PKA-dependent modulation of PIEZO2, we next examined the effect of KT5720 and 8-Br-cAMP treatment on PIEZO2 mutants that completely lack individual IDRs (IDR1^del^–IDR7^del^), which we had previously generated and characterized (33). All mutants, except for IDR5^del^, were modulated by PKA activation (Figs. 4, *C*–*F* and S4), which was surprising at first glance because IDR5 is one of two IDRs that do not contain a single putative PKA phosphorylation site (Table S1 and Fig. 4*A*). In this context, it is, however, important to note that we had previously provided evidence suggesting that PIEZO2 is predominantly activated by cytoskeleton-transmitted forces in the poking assay, whereas it is activated by membrane stretch–derived force in pressure-clamp recordings (33). Most importantly, we had shown that IDR5 is required for the former—hence, the small poking-evoked IDR5^del^ currents—and had hypothesized that the small residual poking-evoked IDR5^del^ currents are most likely activated by membrane stretch, which inevitably also occurs when poking a cell.

Accordingly, the observation that IDR5^del^ is not modulated by PKA is consistent with the finding that PKA does not modulate stretch-evoked PIEZO2 currents (Fig. 2) but does not provide insights into the underlying molecular mechanism.

### Membrane trafficking or changes in PIEZO2 cluster size and density are not involved in PKA-dependent modulation

**Figure 5:**
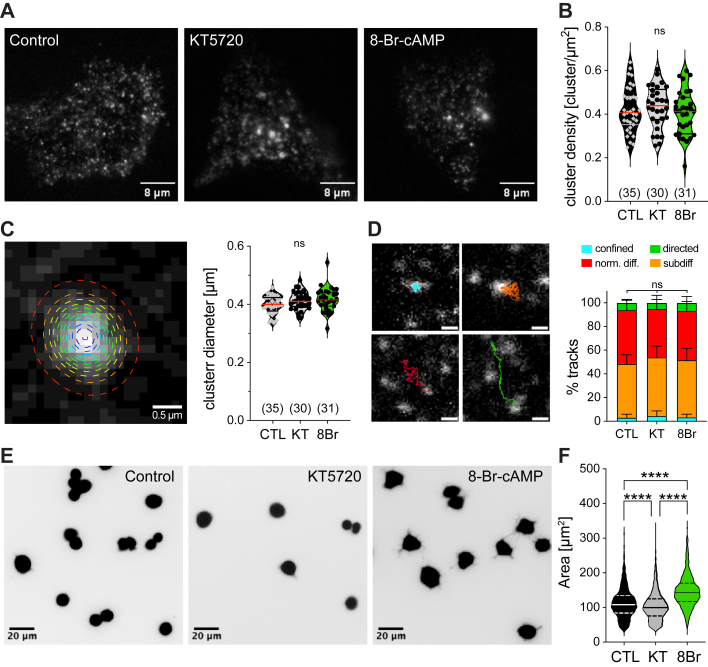
**PIEZO2 cluster size and density is not affected by PKA modulation.***A*, representative TIRF images of N2A-P1KO cells transfected with PIEZO2mScarlet and incubated without (*left*, untreated) or with the PKA inhibitor KT5720 (*middle*) and the PKA activator 8-Br-cAMP (*right*). Scale bar is indicated in the images. *B*, average cluster densities of PIEZO2mScarlet in cells untreated (CTL, *black* or treated with KT5720 (*gray*) or 8-Br-cAMP (*green*). Data are presented as the mean ± SD with individual values. Number of cells is indicated in the graph. Comparison with one-way ANOVA, *p* = 0.5705. *C*, close-up view of the Gaussian fit of a PIEZO2mScarlet cluster (*left*) and average cluster size (in micrometer) per cell (*right*) and per treatment condition. Data are presented as the mean ± SD with individual values. Number of cells is indicated in the graph. Overall cluster number for CTL N = 3623, KT5720 N = 3792, and 8Br N = 5028. Scale bar is indicated in the image. Comparison with one-way ANOVA, *p* = 0.1066. See also Fig. S3. *D*, close-up view and representative examples (*left*) of the four different trajectories observed for PIEZO2mScarlet clusters: confined (*cyan*, *top left*), subdiffusion (*orange*, *top right*), normal diffusion (*red*, *bottom left*), and directed (*green*, *bottom right*). Average proportion of the four defined PIEZO2 cluster trajectories per cell (*right*). Data are presented as the mean ± SD. Number of cells are identical to the ones in *B* and *C*. Overall track numbers are identical to those in Fig. S3*B*. Scale bar represents 1 μm. Comparison with Kruskal–Wallis test, confined *p* = 0.4990, normal diffusion *p* = 0.1799, directed *p* = 0.5502, and subdiffusion *p* = 0.2792. *E*, representative fluorescent images (*inverted*) of N2A-P1KO cells untreated (control, *left*) or treated with the PKA inhibitor KT5720 (KT, *middle*) and the PKA activator 8-Br-cAMP (8Br, *right*). Scale bar is indicated in the images. *F*, quantification of the N2A-P1KO cell area (in μm^2^) incubated with the different PKA-modulated conditions. Data are presented as violin plot with the median value and the 25th and 75th quartile. Number of cells per group is CTL N = 1204, KT5720 N = 1637, and 8Br N = 939. Comparison with Kruskal–Wallis test *p* < 0.0001 and Dunn’s post-test, *p* = 0.00000065 CTL *versus* KT5720, *p* < 0.0001 KT *versus* 8Br, and *p* < 0.0001 CTL *versus* 8Br. 8-Br-cAMP, 8-bromo-cyclic-AMP; TIRF, total internal reflection microscopy.

Since neither mutation of PKA sites on individual IRDs nor deletion of the entire IDRs that contain PKA sites altered PKA-dependent modulation of PIEZO2, we next considered altered membrane expression as a possible mechanism underlying PKA-dependent modulation. PIEZO channels form clusters in the plasma membrane both at endogenous expression levels and when heterologously expressed at high levels (33, 40, 41). Hence, to quantify membrane expression levels, we analyzed the density and size of PIEZO2 clusters of untreated, KT5720-, and 8-Br-cAMP-treated N2a cells expressing an mScarlet-tagged version of PIEZO2 using total internal reflection microscopy (TIRF). This analysis revealed that neither cluster density (Fig. 5, *A* and *B*) nor cluster size (Fig. 5, *C* and *D*) was altered by PKA activity, indicating that the total number of PIEZO2 channels in the plasma membrane was the same in all three conditions. We had previously shown that PIEZO2 clusters exhibit four different modes of lateral movement in the plasma membrane—that is, directed movement, normal diffusion, subdiffusion, and confined movement (Fig. 5*D* and (33)). Although it is unclear if the channels that are differentially sensitive to mechanical stimulation have different movement modes, it is tempting to speculate that channels that diffuse quickly—that is, normal diffusion or directed movement—are less efficiently activated by cytoskeleton-transmitted forces as they do not seem to be tightly attached to the cytoskeleton. Hence, we also tracked the movements of PIEZO2 clusters using TIRF time-lapse imaging but found no difference between the proportions of clusters in the four movement modes in the three treatment conditions (Fig. 5*D*). We did, however, observe a small, yet significant, increase in the diffusion speed between 8-Br-cAMP-treated cells compared with KT5720-treated cells, in all four movement modes (Fig. S3, *A*–*F*). When analyzing the TIRF images, we noticed that 8-Br-cAMP-treated cells appeared to be bigger than untreated and KT5720-treated cells. To corroborate this observation on a larger sample, we quantified the cell sizes of untreated, KT5720-, and 8-Br-cAMP-treated N2a cells. This analysis showed that 8-Br-cAMP-treated cells were indeed significantly bigger than untreated and KT5720-treated cells (Fig. 5, *E* and *F*). However, considering that 8-Br-cAMP-treated cells exhibit PIEZO2 currents that are approximately five times bigger than the currents of KT5720-treated cells (Figs. 1, 3 and 4), it seems highly unlikely that a small increase in cell surface area by 50% accounts for such large increase in current amplitude.

#### PIEZO1 is insensitive to changes in PKA activity

**Figure 6:**
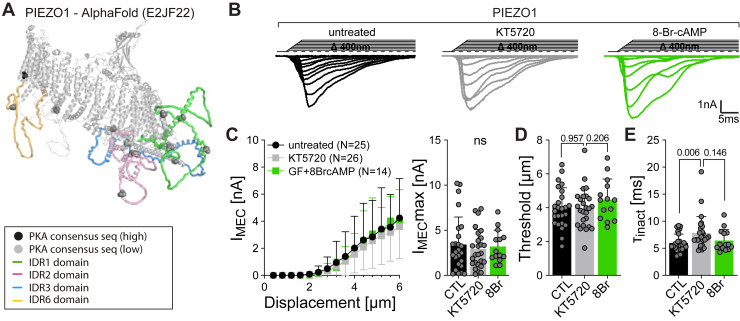
**PIEZO1 mechanically activated currents evoked by membrane indentation are not affected by PKA modulation.***A*, side view of the full-length mouse PIEZO1 AlphaFold structure (E2JF22) with the intracellular disordered loops (*colored domains*) that contained the predicted high (*black sphere*) and low (*gray sphere*) score PKA phosphorylation sites. See also Table S2. *B*, representative example traces from PIEZO1 untreated (*black*, *left*) or treated with KT5720 (*gray*, *middle*) and 8-Br-cAMP (*green*, *right*). *C*, displacement–response curves (*left*) and scatter plot of the maximal (*right*) peak current amplitudes of PIEZO1 untreated (*black*) and treated with PKA inhibitor KT5720 (*gray*) or activator 8-Br-cAMP (*green*). Data are presented as the mean ± SD. Number of cells per group is indicated in the legend. *D*, mechanical activation thresholds from PIEZO1-treated and -untreated cells. Data are presented as the mean ± SD with individual values. Number of cells are identical to (*B*). Comparison with one-way ANOVA, *p* = 0.3858. *E*, inactivation time constants (τ_inact_) of PIEZO1-treated and -untreated cells. Data are presented as the mean ± SD with individual values. Number of cells are identical to (*B*). Comparison with Kruskal–Wallis test, *p* = 0.027 and Dunn’s post-test, *p* = 0.0266 CTL *versus* KT5720. 8-Br-cAMP, 8-bromo-cyclic-AMP.

As mentioned earlier, force transmission *via* the cytoskeleton appears to play an important role in PIEZO channel activation. Thus, it was shown that disruption of the cytoskeleton significantly reduces the amplitudes of poking-evoked PIEZO2 currents (15, 33, 42). Moreover, it was shown that changes in cellular elasticity reduce the mechanosensitivity of sensory neurons that express PIEZO2 (17, 43). Since PKA has been implicated in the remodeling of the cytoskeleton (44), it is conceivable that PKA indirectly facilitates channel activation by altering cytoskeleton stiffness. To test if this or another indirect mechanism is involved in PKA-dependent modulation of PIEZO2, we tested if PIEZO1, which is also sensitive to perturbations of the cytoskeleton (40, 45), but only contains putative PKA phosphorylation sites with a very low prediction score (Table S2 and Fig. 6*A*), is modulated by activation or inhibition of PKA. Interestingly, poking-evoked PIEZO1 currents recorded from untreated, KT5720-treated, and 8-Br-cAMP-treated cells were indistinguishable from one another with regard to current amplitude and mechanical activation threshold (Fig. 6, *B*–*D*), though there was a small difference between the inactivation time constant of untreated and KT5720-treated cells (Fig. 6*E*). In summary, however, the data suggested that PKA does not seem to affect cytoskeleton stiffness or the overall cell elasticity in a way that could explain the strong modulation of poking-evoked PIEZO2 currents.

#### Substitution of all putative PKA sites on IDR1, 2, 3, and 6 abrogates PKA-dependent modulation of PIEZO2

**Figure 7:**
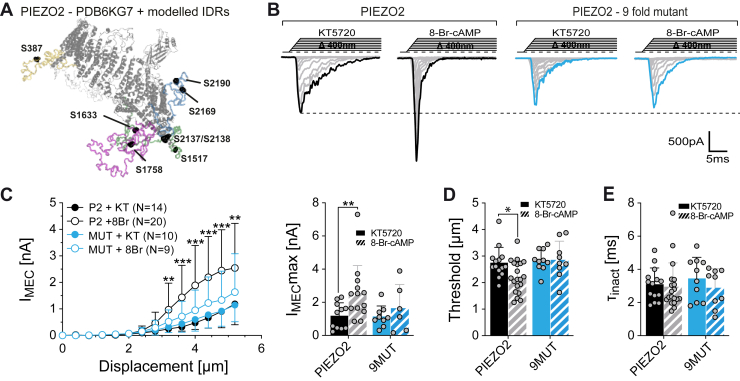
**Simultaneous disruption of all the major predicted PKA sites of PIEZO2 disrupts PKA modulation of mechanically activated currents evoked by membrane indentation.***A*, side view of one protomer of the mouse PIEZO2 structure (Protein Data Bank ID: 6KG7) with the modeled intracellular disordered loops that contained the predicted high score PKA phosphorylation sites that are simultaneously mutated to alanine (*black spheres*). *B*, representative example traces from PIEZO2 (*left*, *black*) and the ninefold mutant (9MUT, *right*) treated with PKA inhibitor KT5720 or with the PKA activator 8-Br-cAMP. *C*, displacement–response curves (*left*) and scatter plot of the maximal (*right*) peak current amplitudes of PIEZO2 (*black*) and PIEZO2-9MUT (*blue*), treated with PKA inhibitor KT5720 or activator 8-Br-cAMP. Data are presented as the mean ± SD. Number of cells per group is indicated in the legend. Comparison with Mann–Whitney test, *p* < 0.05∗, *p* < 0.001∗∗, *p* < 0.0001∗∗∗ PIEZO2 KT5720 *versus* 8Br. *D*, mechanical activation thresholds from PIEZO2 and PIEZO2-9MUT-treated cells with KT5720 (*filled bars*) and 8Br (*dashed bars*). Data are presented as the mean ± SD with individual values. Number of cells are identical to (*C*). Comparison with unpaired *t* test, *p* = 0.0166, PIEZO2 KT5720 *versus* 8Br, *p* = 0.9753 9MUT KT5720 *versus* 8Br. *E*, inactivation time constants (τ_inact_) of PIEZO2- and PIEZO2-9MUT-treated cells with KT5720 (*filled bars*) and 8Br (*dashed bars*). Data are presented as the mean ± SD with individual values. Number of cells are identical to (*C*). Comparison with Mann–Whitney test, *p* = 0.3579, PIEZO2 KT5720 *versus* 8Br, *p* = 0.3562 9MUT KT5720 *versus* 8Br. 8-Br-cAMP, 8-bromo-cyclic-AMP.

Since neither channel trafficking (Fig. 5) nor indirect effects such as stiffening of the cytoskeleton (Fig. 6) appeared to be involved in PKA-dependent modulation, we reconsidered the role of direct phosphorylation and asked if multiple putative PKA sites on different IDRs might be required. To this end, we generated a PIEZO2 mutant in which the serines of all nine putative PKA sites on IDR1, 2, 3, and 6 were substituted by alanines (S387, S412, S1517, S1633, S1758, S2137, S2138, S216, and S2190A). Strikingly, the poking-evoked currents of this nonuple mutant, recorded in the presence of KT5720 and 8-Br-cAMP, respectively, were indistinguishable from one another with respect to amplitudes (Fig. 7, *A* and *B*) and mechanical activation thresholds (KT5720, 2.85 ± 0.42 μm *versus* 8-Br-cAMP, 2.85 ± 0.71 μm; mean ± SD, Fig. 7*C*), whereas wildtype PIEZO2 currents recorded in the same session were significantly larger and had lower mechanical activation thresholds in the presence of 8-Br-cAMP (threshold KT5720, 2.74 ± 0.58 μm *versus* 8-Br-cAMP, 2.22 ± 0.61 μm, mean ± SD; Fig. 7, *A*–*C*). As found for wildtype PIEZO2 and all other mutants, the inactivation kinetics were not altered by PKA activation. Hence, our data suggest that multiple sites located on distant intracellular domains are required for PKA-dependent modulation of PIEZO2.

To rule out the possibility that the multiple alanine substitutions induce abnormal folding in the normally disordered IDRs and thus indirectly affect PKA-dependent modulation of PIEZO2 by altering possible intraprotein or interprotein interactions, we next analyzed the levels of intrinsic disorder of PIEZO2 and the nonuple PKA-site mutant using the disorder prediction tool IUPred2A (46). This analysis, however, showed that the level of intrinsic disorder is not altered by the alanine substitutions (Fig. S5), which supports the idea that the lack of PKA-dependent modulation of the nonuple mutant results from a lack of phosphorylation rather than indirect effect.

## Discussion

Many proinflammatory mediators, such as prostaglandin E_2_, serotonin, and bradykinin, which induce mechanical pain hypersensitivity, exert their actions *via* G_s_-coupled receptors that activate PKA (31). Here, we show that PKA potentiates PIEZO2-mediated mechanotransduction currents and identify multiple putative PKA sites on different intracellular PIEZO2 domains to trigger this effect. Importantly, we also found that PKA only modulates PIEZO2-mediated currents that are evoked by poking the cell surface in whole-cell patch-clamp recordings but not currents recorded with the pressure-clamp technique, which suggests that the two types of mechanical stimuli activate PIEZO2 *via* different mechanisms that can be modulated independent of one another.

Consistent with a previous report by Dubin *et al.* (30), we found that activation of PKA causes a remarkable increase in the amplitudes of poking-evoked PIEZO2 currents (Fig. 1, *B* and *C*). Regarding the mechanistic basis of this effect, our data suggest that the PKA-induced increase in PIEZO2 current amplitudes mainly results from a change in the mechanical activation threshold (Fig. 1*E*) and does not involve PKA-dependent changes in PIEZO2 membrane expression, single channel conductance, or unspecific effects on cell mechanics that might indirectly alter mechanosensitivity. The PIEZO2 membrane expression levels were determined by measuring the density and size of mScarlet-tagged PIEZO2 clusters with TIRF microscopy (Fig. 5). Because of limitations in fluorescence intensity of the mScarlet tag and the spatial resolution of the microscope, this approach, however, precludes the detection of individual channels that are not part of bigger clusters but can nevertheless function as mechanosensors (47). An increase in the number of such “undetectable” channels in the plasma membrane seems, however, unlikely to contribute to the potentiation of the poking-evoked PIEZO2 currents by PKA, because an increased channel number would inevitably also increase the proportion of responding patches and the current responses observed in pressure-clamp recordings, which was not the case (Fig. 2, *A*–*C*). With regard to the influence of cell mechanics on PIEZO channel sensitivity, it is well acknowledged that PIEZO1 and PIEZO2 are equally sensitive to perturbations of cytoskeletal integrity. Thus, pharmacological or genetic disruption of the cytoskeleton inhibits both poking-evoked PIEZO1 and PIEZO2 currents, whereas stiffening of the cells facilitates PIEZO activation (15, 17, 33, 40, 42, 43, 45). PKA, which has previously been implicated in cytoskeleton remodeling (44), however, only modulated PIEZO2 but not PIEZO1 currents (Figs. 1 and 6), suggesting that unspecific effects on cell mechanics that could indirectly facilitate PIEZO2 activation are not involved in PKA-dependent modulation of PIEZO2. Hence, in summary, our data suggest that the PKA-dependent increase in poking-evoked current amplitudes mainly results from a reduction in the mechanical activation threshold of PIEZO2.

With regard to the molecular mechanism, our data suggest that phosphorylation of multiple serines on different intracellular intrinsically disordered domains is required for the sensitization of PIEZO2. Thus, removal of all putative PKA sites from individual intracellular domains, by substituting serines with alanines, had no detectable effect on the PKA sensitivity of PIEZO2 (Fig. 3), but mutating all nine PKA sites on IDR1, 2, 3, and 6 simultaneously completely abolished PKA-dependent modulation (Fig. 7). Whether all nine serines that we had mutated in the nonuple mutant are equally important—that is, whether all or just some of them are required—for PKA-dependent modulation is, however, unclear. Considering that 490 different *n*-tuple mutants (all possible double, triple, quadruple, etc. mutants) would have had to be generated and experimentally tested to definitively answer this question, we did, however, not further address this question using site-directed mutagenesis. Instead, we performed mass spectrometry analysis of PIEZO2 expressed in N2a cells treated with either PKA inhibitor or PKA activator. However, despite several attempts and using different approaches to enrich phosphorylated PIEZO2 peptides in the protein samples such as purification of hemagglutinin (HA)-tagged PIEZO2 with anti-HA agarose beads and use of a TiO_2_ phosphopeptide enrichment kit (*i.e.*, approaches that had previously been used to map phosphorylated residues in PIEZO1 (48)), the peptides that were identified with mass spectrometry only covered around 10% of the total PIEZO2 protein sequence and did not include the sequences of interest (data are available *via* ProteomeXchange with identifier PXD041372). Hence, our data solely show that PKA sites on more than one IDR are required for PKA-dependent modulation of PIEZO2, but it does not allow a definitive conclusion regarding which of the nine putative PKA sites are involved.

Regarding the question as to whether phosphorylation *per se* is sufficient to sensitize PIEZO2 or whether it solely creates a binding site for a protein interaction partner that eventually sensitizes the channel, we can, however, only speculate. Phosphorylation of intrinsically disordered proteins can produce changes in structural properties of the protein (49), thereby altering important biological functions by promoting or inhibiting interaction with other proteins. Likewise, however, substituting serines with alanines could theoretically have the same effect. Thus, since we did not directly demonstrate that the putative PKA sites are indeed phosphorylated upon PKA activation (*e.g.*, with mass spectrometry), we cannot definitively rule out that the alanine substitutions abolish PKA-dependent modulation *via* indirect effects, such as altered folding or altered protein–protein interactions. The former seems unlikely, because the level of disorder does not seem to be altered in the ninefold mutant (Fig. S5), but additional studies will be required to definitively clarify this question.

In addition to unraveling the mechanism of PKA-depending modulation of PIEZO2, our data also provide interesting conceptual insights regarding the gating mechanism of PIEZO2. Ever since PIEZOs were discovered in 2010 (50), there has been an ongoing debate about whether they are activated by force-from-lipids gating (*i.e.*, conformational changes caused by membrane stretch–induced changes in lipid bilayer asymmetry) or by force-from-filament gating (*i.e.*, transmission of forces from the cytoskeleton or the extracellular matrix to the channel by molecular tethers) (51, 52, 53). Proponents of the force-from-lipids model have used the observation that PIEZO1 is sensitive to membrane stretch in lipid droplets and membrane blebs that are completely or largely devoid of a cytoskeleton (40, 54) to argue that force-from-lipid model is the unifying mechanism that gates PIEZOs and that the role of the cytoskeleton is merely to transmit forces to the membrane (51, 53, 55). However, there is now increasing evidence supporting the idea that force-from-lipid and force-from-filament gating exist side by side and that PIEZOs are in fact polymodal mechanosensors that detect different types of mechanical stimuli *via* distinct mechanotransduction mechanisms. Thus, Wang *et al.* (34) have shown that PIEZOs indeed physically interact with the cytoskeleton *via* the transmembrane protein E-cadherin, and we have found that PIEZO2 in sensory neurons requires the extracellular tether-like protein USH2A for normal function (56). Moreover, using conformation-sensitive fluorescent probes that were genetically inserted into different locations of PIEZO1, Ozkan *et al.* (32) have shown that different types of mechanical stimuli cause conformational changes in different regions of PIEZO1. Finally, we have recently shown that deletion of IDR5 significantly attenuates poking-evoked PIEZO2 currents and renders them completely insensitive to perturbations of the cytoskeleton, whereas it does not affect stretch-evoked currents (33). Together, these studies indicated that different types of mechanical stimuli engage different protein domains and force-transmission pathways to activate PIEZO channels. Moreover, they suggested that poking-evoked currents are predominantly activated by force-from-filament model, whereas pressure-induced membrane stretch–evoked currents appear to be activated by force-from-lipid model. Our current data strongly support this dual mechanogating hypothesis by showing that poking and stretch-evoked PIEZO2 currents can be modulated independent of one another by post-translation modification of the channel (Figs. 1 and 2). Considering that PKA does not modulate stretch-evoked PIEZO2 currents, our observation that PKA fails to modulate IDR5^del^ currents (Fig. 4) also supports our previous hypothesis suggesting that IDR5 is required for force-from-filament gating and that poking-evoked IDR5^del^ currents are thus predominantly activated by membrane stretch (*i.e.*, force-from-lipids model) (33).

In summary, our current data together with the aforementioned studies support the idea that PIEZO2 can be activated by two distinct mechanogating mechanisms. Since it is still unclear to which extent the different mechanogating mechanisms contribute to PIEZO2 activation in different cellular contexts, this observation calls for caution when interpreting data obtained with only a single experimental mechanostimulation technique (*i.e.*, poking *versus* stretch) with regard to their physiological relevance. Moreover, the observation that PKA-dependent modulation requires phosphorylation of multiple residues on multiple IDRs together with the observation that PKA-dependent modulation is modality and isoform specific provides an invaluable mechanistic framework for future studies that aim at unraveling the physiological relevance of fine-tuning mechanosensitivity by PKA.

## Experimental procedures

### Cell culture, transfection, and drug treatment

Mouse neuroblastoma Neuro-2a PIEZO1-Knockout cell line (N2a-P1KO) was generated from Neuro-2a American Type Culture Collection CCL-131 and was a gift from G.R. Lewin (35). Cells were grown in Dulbecco’s modified Eagle's medium and optimal minimal essential medium (1:1 mixture), supplemented with 10% fetal bovine serum, 2 mM l-glutamine, and 1% penicillin/streptomycin (all from Thermo Fisher). For TIRF imaging experiments, medium without phenol red was used. N2a-P1KO cells were cultured at 37 °C with 5% CO_2_. Cells were seeded on poly-l-lysine (PLL; Sigma)–coated and acid-washed glass coverslips (for patch-clamp recordings), PLL-coated 12-well plate (cell size measurement), or PLL-coated 35 mm glass-bottom dishes (MatTek High Precision 1.5 Coverslip, TIRF microscopy live imaging). N2A-P1KO cells were transfected 1 or 2 days after plating using polyethylenimine (PEI, Linear PEI 25K; Polysciences). For one coverslip or a single well, 7 μl of a 360 μg/ml PEI solution is mixed with 9 μl PBS. Plasmid DNA is diluted in 20 μl PBS (0.6 μg/coverslip), and then, the 16 μl PEI-PBS solution is added. After at least 5 min of incubation, the DNA–PEI mix is added drop by drop in one well and mixed by gentle swirling. For a 35 mm dish, 1.5 μg DNA is used, and PBS–PEI volumes are adjusted accordingly. About 24 h later, the medium is replaced by fresh one. Cells are then used within 24 h to 48 h.

To investigate PKA activity, N2a-P1KO cells were incubated the day before the experiments (patch clamp or imaging) with PKA inhibitor KT5720 (Sigma) and PKC inhibitor GF109203X (Sigma), both dissolved in dimethyl sulfoxide and used at a final concentration of 1 μM. The two drugs were kept in the medium the day of the experiment for PKA-inhibited experimental groups. PKA activation was done at least 1 h before the experiments and was achieved by treating previously PKA- and PKC-inhibited cells with fresh serum-free medium containing 300 μM of the PKA activator 8-Br-cAMP (Sigma) and 1 μM of the PKC inhibitor GF109203X. Respective drugs were also added in the extracellular buffer during patch-clamp and imaging experiments.

### Constructs and generation of PIEZO2 mutants

A PIEZO2-HA-IRES-GFP plasmid generated previously (36) from mouse piezo2-pSPORT6 plasmid (gift from A. Patapoutian), having at the C terminus an HA-IRES-GFP sequence, was used as the initial template to generate all the constructs of the present study using a similar strategy as described in an earlier study (33, 36). PIEZO2 IDR deletion mutants and PIEZO2mScarlet were generated and extensively characterized in a previous study (33). PKA site point mutants were generated by PCR using specific primers (Sigma) using KAPA HiFi polymerase (Roche). Double (S387A/S412A and S1517A/S1633A) and quadruple (S2137A/S2138/S2169A/S2190A) mutants were generated sequentially, using single (S387A, S1517A) or double (S2137A/S2138A) mutants as a template. The PIEZO2-HA-IRES-GFP 9-fold PKA mutant (S387A/S412A, S1517A/S1633A, S1758A, S2137A/S2138/S2169A/S2190A, PKA-All) was generated using the S2137A/S2138/S2169A/S2190A quadruple mutant as a template. Three PCR fragments containing the other PKA mutations were added with homologous recombination (NEBuilder HiFi; New England Biolabs). PCRs were digested with DpnI (New England Biolabs; 37 °C, 1 h) and column purified with standard kits (NucleoSpin from Macherey-Nagel or PureLink from Invitrogen) before being transformed in electrocompetent Stbl4 bacteria (Invitrogen) and grown at 30 °C for 48 h. Selected clones were entirely sequenced to ensure that no other mutation was present.

### Electrophysiology

Mechanically activated currents were recorded at room temperature using EPC10 amplifier with Patchmaster software (HEKA Elektronik). Borosilicate patch pipettes (2–5 MΩ for whole cell, 3–7 MΩ for cell-attached single channel) were pulled with a P-97 Flaming-Brown puller (Sutter Instrument Company). For whole-cell patch clamp, intracellular buffer contained the following (in millimolar): 125 potassium gluconate, 7 KCl, 1 MgCl_2_, 1 CaCl_2_, 4 EGTA, 10 Hepes, 2 GTP, and 2 ATP (pH 7.3 with KOH). For cell-attached single channel: 130 NaCl, 5 KCl, 1 MgCl_2_, 1 CaCl_2_, 10 Hepes, and 10 tetraethylammoniumchloride (pH 7.3 with NaOH). The control bath solution for whole cell contained the following (in millimolar): 140 NaCl, 4 KCl, 1 MgCl_2_, 2 CaCl_2_, 4 glucose, and 10 Hepes (pH 7.4 with NaOH). For single-channel recordings, the bath solution contained 140 KCl, 1 MgCl_2_, 2 CaCl_2_, 10 glucose, and 10 Hepes (pH 7.4 with KOH). Drugs were added in the extracellular buffer as appropriate and described previously. Cells were held at a holding potential of −60 mV (whole cell) or −100 mV (cell-attached single channel).

Poking-evoked currents were recorded in the whole-cell mode of the patch-clamp technique with a sampling frequency of 200 kHz and filtered with 2.9 kHz low-pass filter. Pipette and membrane capacitance were compensated using the auto function of Patchmaster. Currents were evoked by applying mechanical ramp-and-hold stimuli to the plasma membrane with a fire-polished glass pipette (tip diameter = 2–3 μm) that was positioned opposite to the recording pipette, at an angle of 45° to the surface of the dish and moved with a velocity of 1 μm/ms by a piezo-driven micromanipulator (Nanomotor MM3A; Kleindiek Nanotechnik). Recordings with excessive leak currents, unstable access resistance, and cells that giga seals did not withstand at least seven consecutive mechanical stimuli were excluded from analyses. Mechanical thresholds of PIEZO2 currents were determined by measuring the latency between the onset of the mechanical stimulus and the onset of the mechanically activated current. Current onset was defined as the point in which the current significantly differed from the baseline (more than six times the SD of the baseline). The membrane displacement at which the current was triggered was then calculated by multiplying the speed at which the mechanical probe moved (1 μm/ms) with the latency. The inactivation time constants (τ_inact_) were measured by fitting the mechanically activated currents with a single exponential function (C1 + C2∗exp(−(*t* − *t*0)/τ_inact_)), where C1 and C2 are constants, *t* is time and τ_inact_ is the inactivation time constant.

Stretch-evoked currents were recorded in the cell-attached configuration with a sampling frequency of 50 kHz and filter with a 2.9 kHz low-pass filter. Currents were evoked by applying negative pressure stimuli (500 ms duration, −5 mm Hg increments up to −80 mm Hg) to the plasma membrane *via* the patch pipette using the High-Speed Pressure Clamp device (ALA Scientific Instruments). For I/V experiments, pressure stimulus was adjusted on a cell-by-cell basis to optimally evoke single-channel openings. As observed by us and others before (33), repetitive and sustain pressure pulses often made PIEZO2 to produce noninactivating responses, making the determination of a peak current value difficult. Therefore, we calculated the total charge transferred during the pressure stimulus (in pico Coulomb) through the determination of the area under the curve over the 500 ms stimulus. Single-channel amplitudes at a given holding potential (−140 mV to −40 mV, 20 mV steps) were determined as the difference between the peaks of the gaussian fits of the trace histogram over a 500 ms segment. Unitary conductance was determined from the linear regression fits of the I/V plot of individual cells. Recordings with excessive leak currents or unstable baseline were excluded. Recordings that displayed noninactivating responses or unstable openings were not used for I/V analyses.

### TIRF microscopy and live imaging

TIRF imaging was performed on a Nikon Eclipse TiE microscope and with the Roper iLAS2 TIRF module. The objective was an oil immersion Nikon CFI Plan Apo Lambda 100× (numerical aperture = 1.45). A 1.5× magnification lens was added giving a final pixel size of 0.11 μm. The TIRF angle was adjusted manually for every cell if necessary. The camera used was a Photometrics Prime 95B back-illuminated sCMOS, having a resolution of 1200 × 1200 pixels. Cells were illuminated with a 561 nm excitation laser (10% power) and were imaged for 30 s with a frame rate of 10 Hz (approximately 100 ms exposure time per frame). An on-stage incubation chamber (Okolab) was used to adjust temperature (37 °C), CO_2_ concentration (5%), and humidity. N2A-P1KO cells transfected with PIEZO2mScarlet were prepared as described in the previous section, incubated with appropriate drugs, and imaged in phenol red–free medium.

Spot and track analyses were processed as described elsewhere (33). Time-lapse recordings were preprocessed in ImageJ before track analysis, with a bleach correction (histogram matching) (57). PIEZO2mScarlet track analysis was then performed with TrackMate v7.6.1 (58, 59) and the following parameters: DoG detector, blob diameter of 0.7 μm, spot quality filter value of 0.75, simple LAP tracker with a linking distance of 0.5 μm, a gap closing distance of 0.7 μm, and a maximal gap closing frame number of 2. For mean square displacement calculation, only tracks that have a duration of at least 40 frames were considered. Additional track classification was done in ImageJ with TraJClassifier plugin (60). Mean square displacement calculation was then performed in Igor Pro 8 (WaveMetrics). To analyze the diameter of PIEZO cluster and their density per cell, the total number of spots detected by TrackMate on the first frame was used, and the cell area was manually determined in ImageJ. Individual spot diameter calculation was performed in Trackmate by using the refine spot location option and by fitting them with a 2D Gauss function.

Live imaging for N2a-P1KO cell-size determination upon PKA manipulation was performed on an Axiovert135 microscope, with an Olympus Plan C 10× objective and a Sumix SMX-M8x camera. In addition to PKA drugs, cells were incubated in phenol red–free medium with 2 μM CellTracker Green dye (Thermo Fisher) for at least 30 min prior to acquisition. Cells were then washed and incubated again with the appropriate drugs during imaging.

### Modeling and site prediction

To visualize the location of the PKA sites along the PIEZO2 IDRs, a modified version of the PIEZO2 structure (Protein Data Bank ID: 6KG7) was used, as described previously (33): the unresolved IDRs were generated first with SWISS-MODEL, modeled using the full-length PIEZO2 and the existing PIEZO2 cryo-EM structure as template (61). Then, the IDRs were removed from the model and added to the original PIEZO2 structure (Protein Data Bank ID: 6KG7). For PIEZO1, the mouse AlphaFold model (E2JF22) was used (62). All molecular images of PIEZO were generated with PyMOL 2.4.0 (Schrödinger, LLC). PKA site prediction was done with NetPhos 3.1 (38) and GPS 5.0 (39) on the mouse PIEZO2 and PIEZO1 sequence. Conservation/consensus score was determined with a multiple alignment in Jalview of PIEZO2 from different vertebrate species retrieved from Ensembl database (63).

### Data analysis

Results are expressed as means ± SD (unless otherwise noted). Statistical analyses were performed with Excel and Prism 8.0 (GraphPad Software, Inc). Data distribution was systematically evaluated, and following statistical tests were chosen accordingly. Two-tailed tests were systematically used. Patch-clamp data were analyzed with FitMaster (HEKA) and Igor Pro 8. Statistical tests that were used, exact *p* values, and information about the number of replicates/cells are provided in each of the figure or in the corresponding legends. Symbols on graphics (∗ or #) indicate standard *p* value range: ∗*p* < 0.05; ∗∗*p* < 0.01; ∗∗∗*p* < 0.001, and ns (not significant) *p* > 0.05.

## Data availability

Source data files and plasmids generated in this study are available upon request from the corresponding author (s.lechner@uke.de).

## Supporting information

This article contains supporting information.

## Conflict of interest

The authors declare that they have no conflicts of interest with the contents of this article.
