# Supplementary material for: PKA mediates modality-specific modulation of the mechanically gated ion channel PIEZO2
Source: J Biol Chem. 2023 May 4;299(6):104782. doi: 10.1016/j.jbc.2023.104782 (PMC10240422; doi:10.1016/j.jbc.2023.104782)
Supplement: Supplemental Figures S1–S5, Tables S1 and S2 [file mmc1.pdf]

Supporting Information

Title: „ Protein kinase A mediates modality-specific modulation of the mechanically-gated ion channel PIEZO2”

Authors

Irina Schaefer, Clement Verkest, Lucas Vespermann, Thomas Mair, Hannah Voß, Nadja Zeitzschel & Stefan G. Lechner

Content

Figure S1
Figure S2
Figure S3
Figure S4
Figure S5
Table S1
Table S2

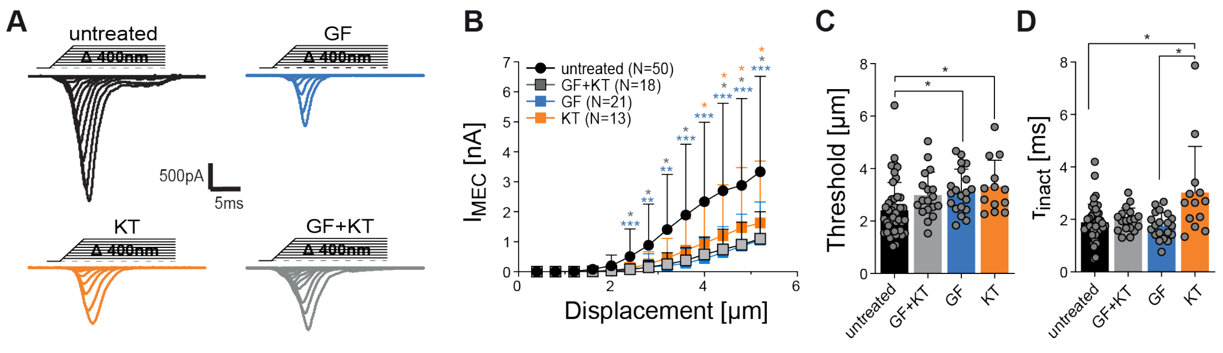

Figure S1. Basal PKA and PKC activity modulated poking-evoked PIEZO2 currents. **(A)** Representative example traces from PIEZO2 currents from untreated cells (top left, black), treated with only the PKC inhibitor GF109203X (top right, blue), with only the PKA inhibitor KT5720 (bottom left, orange) or with both inhibitors (bottom right, grey). **(B)** Displacement-responses curves of peak current amplitudes of PIEZO2 from untreated (black) cells and cells treated with the PKC inhibitor GF109203X (blue), the PKA inhibitor KT5720 (orange) or both (grey). Data are presented as the mean ± SD. Number of cells per group is indicated in the legend. Comparison with Kruskal Wallis and Dunn's post test, $p<0.05$ *, $p<0.001$ **, $p<0.0001$ ***, untreated vs treated. **(C)** Mechanical activation thresholds from PIEZO2 untreated and treated cells. Data are presented as the mean ± SD with individual values. Number of cells are identical to (B). Comparison with Kruskal Wallis test, $p=0.0019$, and with Dunn's posttest, $p=0.0121$ untreated vs GF, $p=0.022$ untreated vs KT. **(D)** Inactivation time constants (τ_{inact}) of PIEZO2 untreated and treated cells. Data are presented as the mean ± SD with individual values. Number of cells are identical to (B). Comparison with Kruskal Wallis test, $p=0.0112$, and with Dunn's posttest, $p=0.029$ untreated vs KT, $p=0.0171$ GF vs KT.

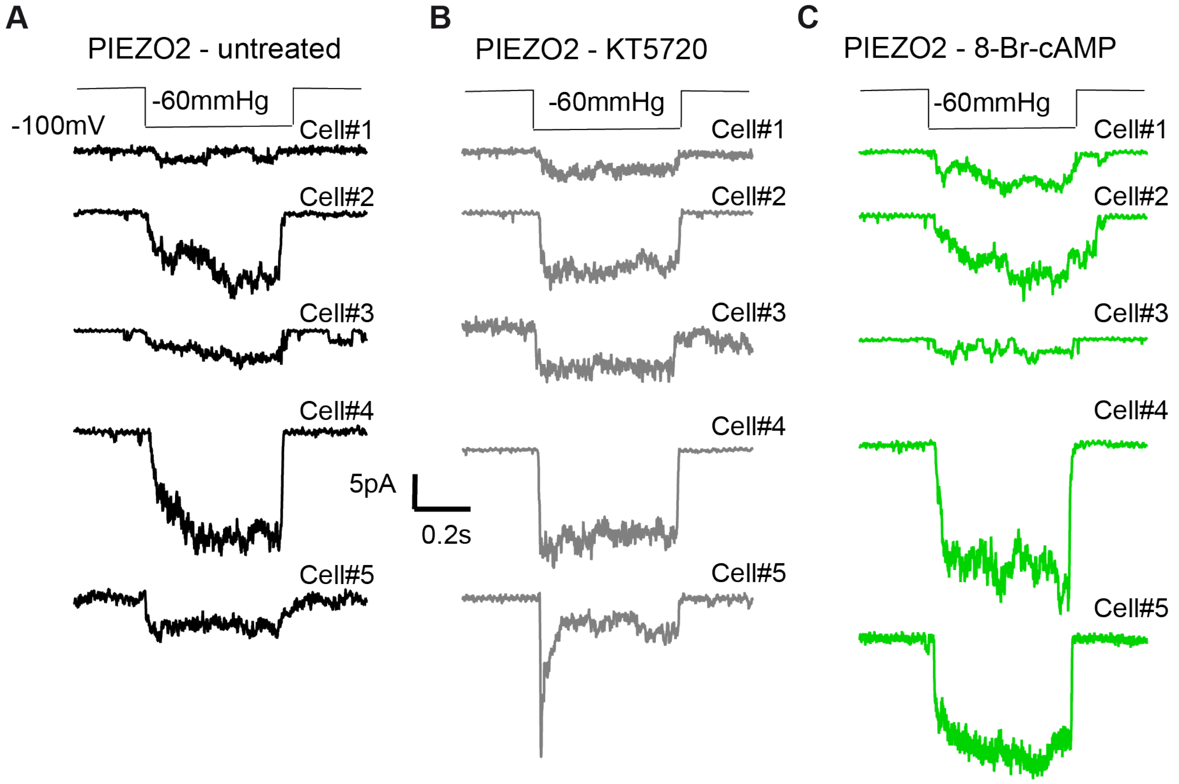

Figure S2. PIEZO2-dependent stretch-activated currents under the different PKA treatment conditions. **(A-C)** Representative PIEZO2 stretch-activated current evoked by a -60mmHg negative pressure pulses applied to N2a-P1KO cell patches (5 different cells per condition) in the absence (black, A) or presence of the PKA inhibitor KT5720 (grey, B) and the PKA activator 8-Br-cAMP (green, C).

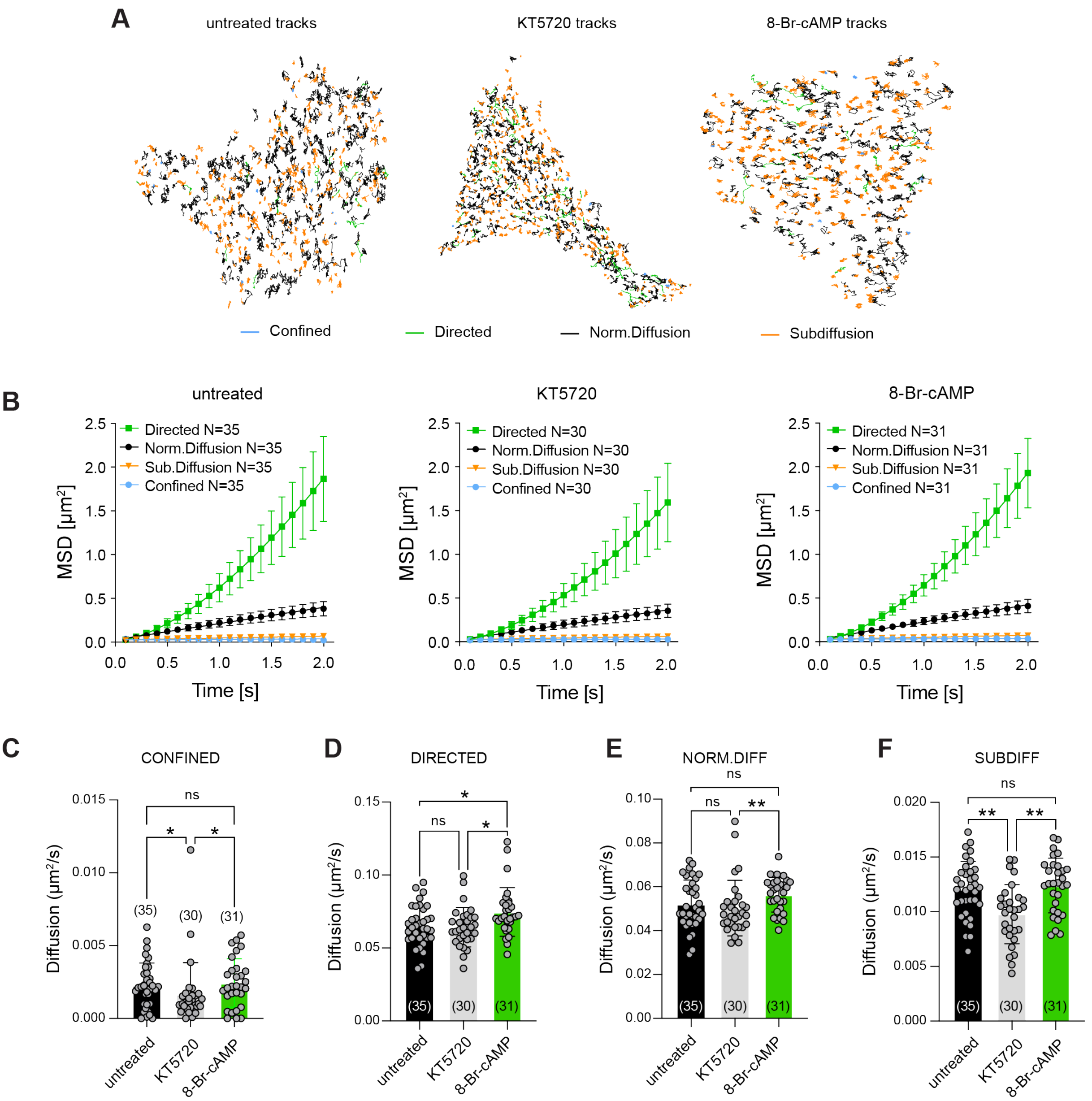

Figure S3. PKA modulation slightly influences PIEZO2mScarlet clusters diffusion. **(A)** Plot of the PIEZO2mScarlet cluster tracks recorded from the 3 different cells shown in figure 5 (A) during 30 seconds, with the different type of trajectories represented. Only tracks that could be followed for 40 frames (4 seconds) are displayed. **(B)** Plots of the Mean-Squared-Displacement (MSD) as a function of lag time for the 4 track categories and for PIEZO2 untreated cells (left) or treated with KT5720 (middle) and 8-Br-cAMP (right). Symbols represent means ± SD from the indicated number of cells. **(C, D, E, F)** Diffusion coefficients of individual PIEZO2mScarlet clusters in the 3 different PKA treatment conditions from the different track categories: confined (C), directed (D), normal diffusion (E) and subdiffusion (F). Data are presented as the mean ± SD with individual values. Number of cells is indicated within each bars. For Directed, comparison with Kruskall Wallis test, p=0.0114, and with Dunn's post test p=0.044 CTL vs 8Br, p=0.018 KT5720 vs 8Br, p>0.999 CTL vs KT5720. For Confined, comparison with Kruskall Wallis test, p=0.0089, and with Dunn's post test p>0.999 CTL vs 8Br, p=0.0177 KT5720 vs 8Br, p>0.0286 CTL vs KT5720. For Normal diffusion, comparison with Kruskall Wallis test, p=0.012, and with Dunn's post test p=0.213 CTL vs 8Br, p=0.009 KT5720 vs 8Br, p=0.0619 CTL vs KT5720. For Subdiffusion, comparison with Kruskall Wallis test, p=0.0007, and with Dunn's post test p>0.999 CTL vs 8Br, p=0.0013 KT5720 vs 8Br, p>0.0065 CTL vs KT5720.

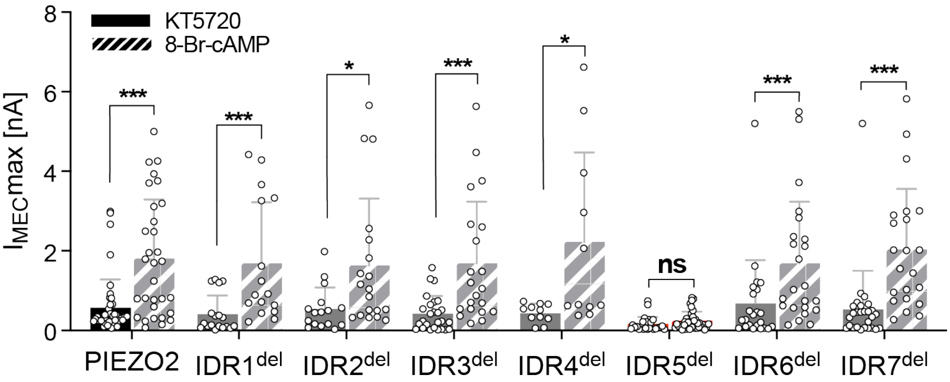

Figure S4. Pairwise comparison of the maximal current amplitudes of mechanically evoked current recorded from N2a-PIEZO1-KO cells transfected with the indicated mutants and treated with either the PKA inhibitor KT5720 (solid bars) or the PKA activator 8-Br-cAMP (cross hatched bars). Bars represent means ± SD and data from individual cells are shown as white dots. Data were compared using multiple Mann-Whitney tests: PIEZO2 (N_{KT} = 44, N_{8Br} = 31; P = 0.000036), IDR1^{del} (N_{KT} = 18, N_{8Br} = 16; P = 0. 0.000376), IDR2^{del} (N_{KT} = 16, N_{8Br} = 19; P = 0.011088), IDR3^{del} (N_{KT} = 26, N_{8Br} = 21; P = 0.000088), IDR4^{del} (N_{KT} = 11, N_{8Br} = 11; P = 0.015769), IDR5^{del} (N_{KT} = 25, N_{8Br} = 36; P = 0.082236), IDR6^{del} (N_{KT} = 23, N_{8Br} =23; P = 0.000917), IDR7^{del} (N_{KT} = 27, N_{8Br} =22; P = 0.0000005).

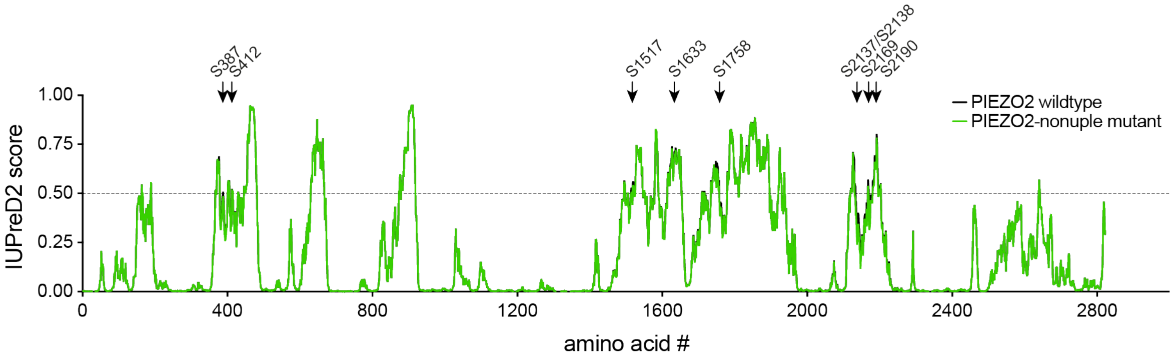

Figure S5. mPIEZO2 (black) and mPIEZO2-9MUT (green) amino acid sequence-based disorder predictions (bottom) determined using IUPRED2A. Positions of the amino acids that were substituted by alanines in the 9-fold mutant are indicated above the graph. Note, alanine substitution does not cause noteworthy changes in the level of intrinsic disorder.

Domain	Amino acid	Consensus sequence	NetPhos3.1 [score]	GPS [score]	Conservation
**TM5-pre-α1**	S206	RRFASVASK	0.599	1.96	46.4
**IDR6**	**S387**	**ERRRSLWYA**	**0.883**	**4.872**	**58.9**
	S412	DYKPSDGLL	0.711		78
	S432	TIHPSLPIE	0.577		64.5
	S472	KREDSEGEG	0.553		30.9
**IDR4**	S856	HPEGSLPDL	0.546		96.4
**IDR3**	**S1517**	**ERMLSLTQE**	**0.845**	**23.296**	**97.4**
	S1594	PPRKSAFQF	0.711		99.5
	**S1633**	**RRKGSGDGP**	**0.796**	**3.667**	**62.4**
	S1652	VKKKSDGPD	0.575	3.803	83.2
**Clasp**	S1719	PTRESIHMY	0.727		91.4
**IDR2**	S1735	LSRESGLDT	0.558		80.4
	**S1758**	**HRMDSLDSR**	**0.699**	**2.446**	**96.6**
	S1764	DSRDSISSC	0.535		90.8
	T1781	SRQSTLDDL	0.621		97.2
	S1906	PSYSKAVSFEHLSFA		3.244	82.2
**IDR1**	**S2137**	**GRRGSSDSL**	**0.857**	**5.408**	**81.3**
	**S2138**	**RRGSSDSLK**	**0.638**	**2.108**	**49.5**
	**S2169**	**RRKRSCSSS**	**0.788**	**24.078**	**91.1**
	S2171	IRRKRSCSSSQISPR		1.972	48.9
	S2180	SQISPRSSFSSNRSK		4.045	65.5
	**S2190**	**SKRGSTSTR**	**0.736**	**3.26**	**93.6**
	T2191	NRSKRGSTSTRNSSQ		3.793	81.2
	S2196	GSTSTRNSSQKGSSV		23.171	94.1
	S2202	NSSQKGSSVLSLKQK		4.044	93.9
**THU9-Anchor-linker**	S2396	FLTKSYNYV	0.501		98.7
**CTD-α1**	T2790	LVRETGELE	0.53		99.2
**CTD-α3**	T2821	IKWTREKTN		2.282	81.4

Table S1. Characteristics of the predicted PKA sites of mouse PIEZO2. The localization within defined PIEZO domain is indicated, as well as the putative phosphorylation amino acid position, the consensus sequence with the phosphosite highlighted, the respective predictive score obtained with NetPhos3.1 and GPS, as well as the conservation (%) of the phosphosite between species. Predictive site detected with both software are highlighted in bold.

Domain	Amino acid	Consensus sequence	NetPhos3.1 [score]	GPS [score]	Conservation
**THU1**	**S51**	PSRH**S**IPGH	**0.657**	**25.876**	**38.3**
**IDR6**	T351	LRKE**T**PRED		5.251	3.6
**THU4-pre-α1**	S573	TLLR**S**LGEL	0.651		46.6
**Beam**	S1362	KYRQ**S**QASR	0.669		13.2
**Clasp**	S1500	QRVL**S**TMQF	0.665		45.1
	S1531	HRTM**S**DVLC	0.703		95.6
**IDR2**	T1574	GPVE**T**RDGP		4.025	8.3
	T1644	TRMR**T**ASEL	0.576		89.9
	S1646	MRTA**S**ELLL	0.674		88.6
**IDR1**	S1864	QGKG**S**IRSK		23.56	4.1
	S1887	TRHI**S**IRFR		5.494	45.6
	S1945	RRLQ**S**FCVS	0.692		28.8
**CTD- α1**	T2516	LVRE**T**RELE	0.616		90.2

Table S2. Characteristics of the predicted PKA sites of mouse PIEZO1. Characteristics of the predicted PKA sites of mouse PIEZO1. The localization within defined PIEZO domain is indicated, as well as the putative phosphorylation amino acid position, the consensus sequence with the phosphosite highlighted, the respective predictive score obtained with NetPhos3.1 and GPS, as well as the conservation (%) of the phosphosite between species. Predictive site detected with both software are highlighted in bold.
